# Supplementary material for: Miro proteins coordinate microtubule‐ and actin‐dependent mitochondrial transport and distribution
Source: EMBO J. 2018 Jan 8;37(3):321–36. doi: 10.15252/embj.201696380 (PMC5793800; doi:10.15252/embj.201696380)
Supplement: Supplementary file 1 — Appendix [file EMBJ-37-321-s001.pdf]

Table of contents

Appendix Supplementary Figure 1

Appendix Supplementary Figure 2

Appendix Supplementary Figure 3

Appendix Supplementary Figure Legends

Appendix Supplementary Methods

Appendix Supplementary References

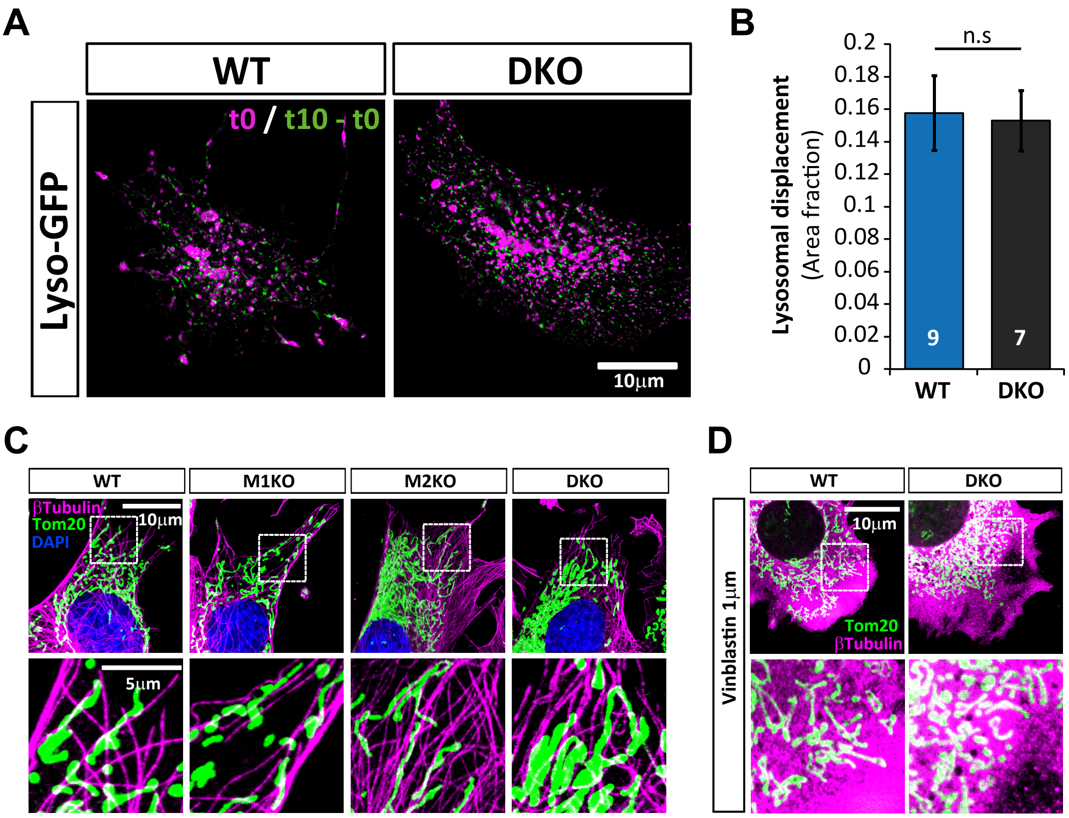

Appendix Supplementary Figure 1

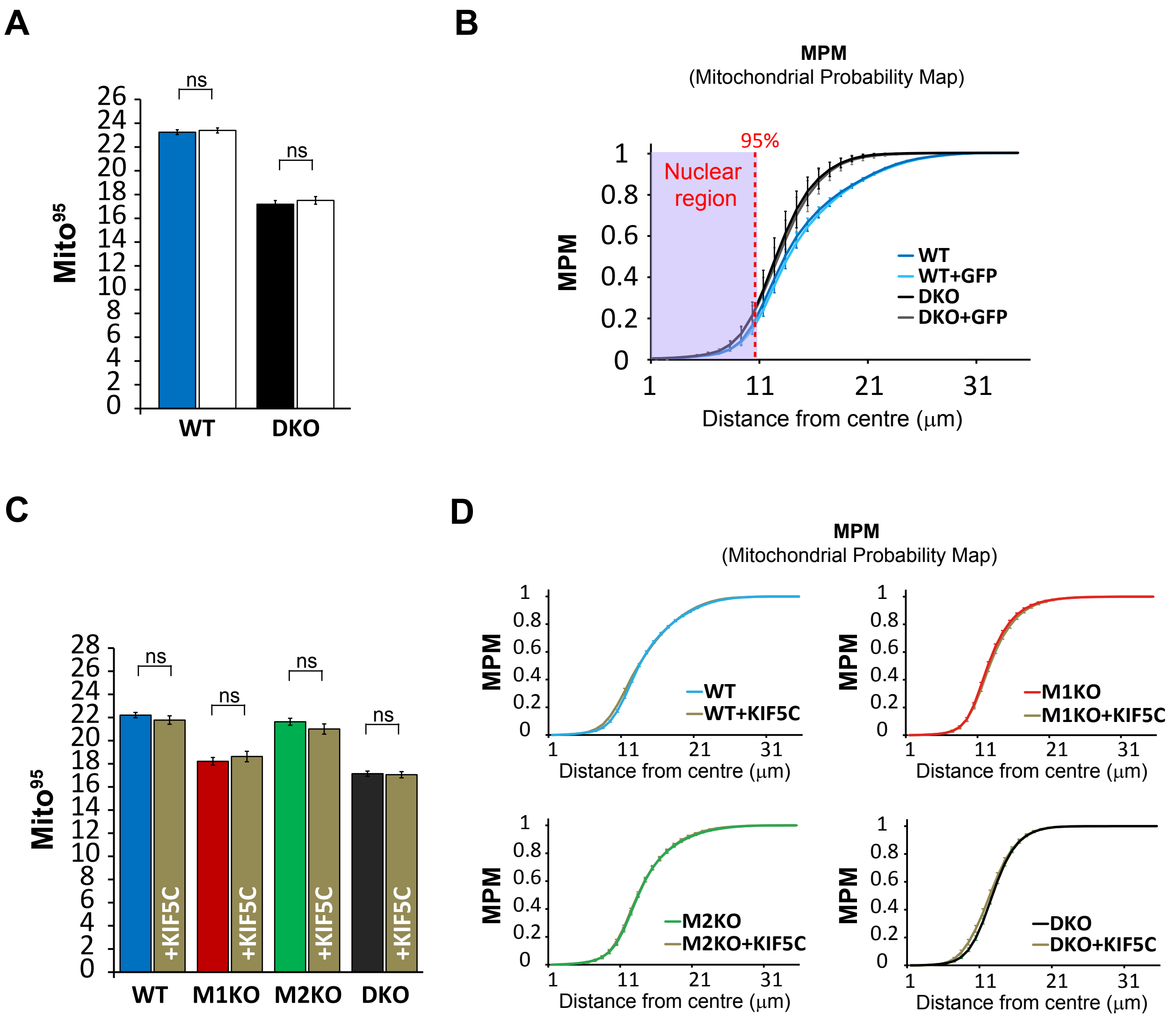

Appendix Supplementary Figure 2

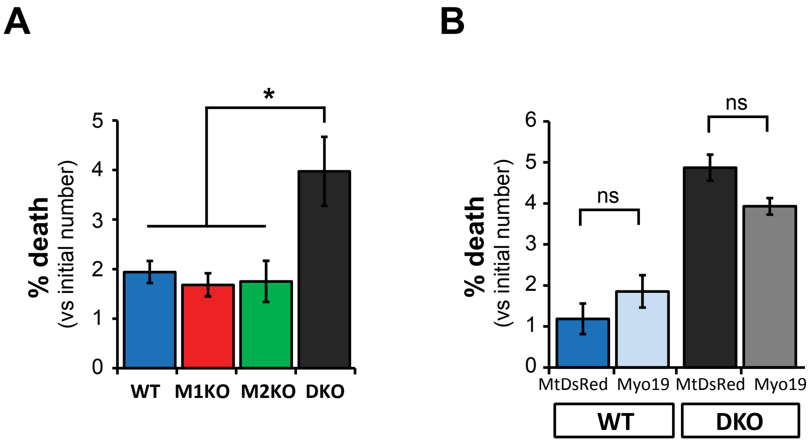

Appendix Supplementary Figure 3

Appendix Supplementary Figure Legends

Appendix Supplementary Figure 1

Lysosomal displacement is not affected by Miro^{DKO} MEF cells. (A) Panels show lysosomal compartment at time=0 (red) and the new area occupied by lysosomes 10 seconds later (lysososmal area at t=10 sec - lysosomal area at t=0) (green). (B) Lysosomal displacement as a fraction of the area newly occupied by lysosomes after 10 seconds of movie. The displacement was calculated by subtracting lysosomal area at two different timepoints separated by 10 seconds between them and normalized to total area at t=0 (t0 / t10 - t0). This displacement was averaged between all pairs of frames separated by 10 seconds (59 pairs over a 61 frame movie) to give a single value per cell. (n= number of cells; values given in graphs; t-test). (C and D) Mitochondria are still associated to the microtubular network in Miro^{DKO} cells. (C) Confocal micrographs showing mitochondrial network aligning with microtubules. (D) Vinblastine treatment induces the disruption of the microtubular network. Error bars represent s.e.m. Significance: *p<0.05, **p<0.01 and ***p<0.001.

Appendix Supplementary Figure 2

(A & B) Overexpression of GFP does not affect mitochondrial distribution. (A) $Mito^{95}$ value or (B) the plotted mitochondrial probability maps (MPM) does not change in either WT or $Miro^{DKO}$ cells when GFP is overexpressed. Data from 60 cells per genotype and condition obtained from 3 independent experiments (n= number of cells; ANOVA-NK). (C & D) Overexpression of KIF5C-myc does not affect mitochondrial distribution. (C) $Mito^{95}$ value or (D) the plotted mitochondrial probability maps (MPM) does not change in any genetic background studied when KIF5Cmyc is overexpressed. Data collected from 3 independent experiments (n= number of cells; WT 52; Miro1KO 39; Miro2KO 48; MiroDKO 51; ANOVA-NK). Each experiment was performed and statistically analysed with all experimental conditions shown in main Figs 4 and 5 applying appropriate corrections for multiple comparisons by ANOVA-NK. Only control and KIF5C are represented in the panels for clarity.

Error bars represent s.e.m. Significance: *$p<0.05$, **$p<0.01$ and ***$p<0.001$.

Appendix Supplementary Figure 3

(A) Percentage of cell death during a 10 hour period from movies taken from the selected genotypes. Values are normalised with the initial number of cells (n= 3 independent experiments; ANOVA-NK). Quantification of cell death (B) in WT and Miro^{DKO} cells transfected with Su9GFP (control) or MtDsRed + GFPMyo19. Values are normalised with the initial number of cells (n= 3 independent experiments; t-test).

Error bars represent s.e.m. Significance: *p<0.05, **p<0.01 and ***p<0.001.

Appendix Supplementary Methods

Animals

The *Rhot1* (MBTN_ EPD0066_2_F01; Allele: *Rhot1*$^{tm1a(EUCOMM)Wtsi}$) and *Rhot2* (MCSF_ EPD0389_5_A05; *Rhot2*$^{tm1(KOMP)Wtsi}$) mice lines were obtained from the Wellcome Trust Sanger Institute (IKMC) (Skarnes et al., 2011) and were already described (Lopez-Domenech et al., 2016). Animals were maintained under controlled conditions (temperature 20 ± 2° C; 12 hour light-dark cycle). Food and water were provided *ad libitum*. Embryos from timed pregnant females were dissected in cold dissection buffer and visually examined prior of genotyping. Embryos at E8.5 were considered viable if they did not show any sign of reabsorption, developmental defect or oedema. Embryos from E10 to E16 were additionally assessed for heart beating. DNA from Yolk sacs was obtained using the QIAamp DNA Mini Kit (QIAgen) and genotyping performed using specific primers for Rho1, Rhot2 and LacZ (Lopez-Domenech et al., 2016).
All experimental procedures were carried out in accordance with institutional animal welfare guidelines and licensed by the UK Home Office in accordance with the Animals (Scientific Procedures) Act 1986. All data involving procedures carried out on in animals is reported in compliance with ARRIVE guidelines (Kilkenny et al., 2010).

DNAs, reagents and antibodies

GFP, Su9GFP, MtdsRed2, Miro1myc and Miro2myc constructs have been already described (Lopez-Domenech et al., 2016). Miro1GFP, Miro2GFP, TRAK1GFP and TRAK2GFP were published elsewhere (Norkett et al., 2016). KIF5Cmyc was already published (Twelvetrees et al., 2010). HA-Ub and Flag-Parkin were used in (Birsa et al., 2014). Homo sapiens myosin XIX (hMyo19) in pDONR223 vector was purchased from Open Biosystems (cDNA clone MGC: 14882 IMAGE:3629870) and subcloned into pEGFP-C1 vector (Clontech). LysoGFP BACMAM 2.0 transduction system was purchased from Invitrogen and used following manufacturer’s instructions. For immunocytochemistry antibodies and reagents were used as follows: rat anti-GFP

(1:1000, Nacalai-Tesque, GF090R); mouse anti-myc was obtained from 9E10 hybridoma line and used as supernatant at 1:100; rabbit anti-Tom20 (1:300, SantaCruz, sc-11415); rabbit anti-Myo19 (1:100, Abcam, ab174286); mouse anti-beta tubulin (1:500, Sigma, T5293); MitoTracker Orange CMTMRos (Invitrogen); Phalloidin-647 ATTO (Sigma). AlexFluor-405, -488, -568 or -647 conjugated secondary antibodies were obtained from Invitrogen. For western blotting antibodies were used as follows: rabbit anti-Miro1 (1:1000, Atlas Antibodies, HPA010687); rabbit anti-actin (1:10000, Sigma, A2066), mouse ApoTrak-cocktail (1:1000, MitoSciences, ab110415); mouse anti-beta tubulin (1:2000, Sigma, T5293); rabbit anti-TOM20 (1:1000, SantaCruz, sc-11415); rabbit anti-COX-IV (1:1000, Abcam, ab16056); mouse anti-PDI (1:1000, BD Biosciences, 610947); rabbit anti-Myo19 (1:500, Abcam, ab174286); rabbit anti-TRAK1 (1:500, Atlas, HPA005853); rabbit anti-TRAK2 (1:500, Atlas, HPA015827); mouse anti-DIC (1:500, Sigma, D5167); mouse anti-p150/GLUED (1:1000, BD Biosciences, 610473). Horseradish peroxidase-conjugated anti-mouse or anti-rabbit IgGs where used at 1:10000 (Rockland).

Cell culture and transfection

SH-SY5Y cells and mouse embryonic fibroblasts (MEFs) were maintained in Dulbecco's Modified Eagle Medium (DMEM) with 4500 mg/L glucose and supplemented with Fetal Bovine Serum (FBS) (COS-7 10%; MEFs 15%), Glutamax 2 mM, Penicillin 120 µg/ml and Streptomycin 200 µg/ml and kept at 37° C and 5% CO_2 atmosphere. For immunocytochemistry experiments MEFs cells were seeded at a density of 50,000 cells / cm^2 on fibronectin coated coverslips. Transfection was carried out using Lipofectamine2000 reagent. For biochemical experiments COS-7 cells were transfected by nucleofection using the AMAXA system. Cycloheximide treatments were performed at 1 μM for the indicated times. Bafilomycin (400 nM) or MG132 (10 μM) were included when indicated. For induction of mitophagy FLAG-Parkin stably

overexpressing SH-SY5Y cells were incubated with 10μM of FCCP for the indicated times as previously published (Birsa et al., 2014).

Respiration experiments

High resolution respirometry was carried out using an Oroboros Oxygraph-2k system (Oroboros Instruments). MEF cells were grown for at least one week to allow for aclimatisation in DMEM medium (15% FBS; 2 mM Glutamax; 120 µg/ml Penicillin and 200 µg/ml Streptomycin) containing either low glucose concentration (1000 mg / l) or galactose (15 mM galactose), in glucose-free medium and were kept at 37° C and 5% CO_2 atmosphere. Cells were trypsinised, washed in PBS and resuspended at a concentration of $1x10^6$ cells / ml in MiR05 medium (EGTA 0.5 mM; $MgCl_2$-$6H_2O$ 3 mM; K-lactobionate 60 mM; Taurine 20 mM; KH_2PO_4 10 mM; HEPES 20 mM; Sucrose 110 mM) containing glucose (1000 ml / l) or galactose (15 mM) as substrate. $2x10^6$ cells were used to measure Routine (R) respiration given as pmol of O_2 consumed per second and 10^6 cells. Oligomycin (2.5 μM) was used to induce uncoupled respiration (LEAK) state, sequential FCCP additions (1 μM steps) to reach maximal respiration capacity (Electron Transport System - ETS); AntimycinA (0.5 μg/ml) was used to analyze residual oxygen consumption (ROX); Ascorbate (20 mM) and TMPD (5 mM) were added to measure maximal capacity of complex IV (C-IV). Results are given as ETS, ratio (R / ETS) and ratio (C-IV / ETS). All experiments were performed with 2 different cell lines per genotype.

Immunocytochemistry and western blotting

MEF cells were fixed when required in 4% PFA in Phosphate Buffered Saline (PBS) for 10 min at RT and rinsed several times in PBS. For immunocytochemistry coverslips were permeabilised in PBS with 0.1% Triton-X100 and incubated 1 hour in blocking solution (1% BSA, 10% fetal bovine serum, 0.2M glycine in PBS with 0.1%Triton-

X100). Primary antibodies where applied in blocking solution at the desired concentration and incubated for 2 hours. AlexaFluor (Invitrogen) secondary antibodies were incubated for one hour at 1:800 in blocking solution. Coverslips were mounted in Mowiol mounting media and kept in the dark at 4°C until imaged. For western blotting 20 μg of protein from MEFs lysates were loaded on 8 - 12% acrylamide gels and transferred to nitrocellulose membranes (GE Healthcare Bio-Sciences) using the Biorrad system. Membranes were blocked in 3% BSA in TBS-T or 4% powder milk in PBS-T for 1-2 hours. Antibodies were incubated in blocking solution (overnight at 4°C for primary antibodies or 1 hour at RT for HRP-conjugated secondary antibodies). Membranes were developed using the ECL-Plus reagent (GE Healthcare Bio-Sciences) and acquired in a chemiluminescence imager coupled to a CCD camera (ImageQuant LAS 4000mini). Densitometric analysis was performed using Quantity-One software (Biorrad). Mitochondrial enrichment was calculated (mitochondria signal / cytoplasmic signal) and normalized to control.

Proximity Ligation Assays (PLA)

MEFs were seeded on fibronectin coated coverslips and grown overnight. Coverslips were fixed at room temperature for 7 minutes using 4% paraformaldehyde in PBS (pH 7.5) supplemented with 4% sucrose, washed 3 times in PBS, and blocked for 10 minutes in blocking buffer (10% Horse Serum, 5 mg/ml BSA, and 0.2% Triton in PBS). Coverslips were then incubated for 1h at room temperature with primary antibodies in blocking buffer (1:250 Mouse anti-Mitofusin 1 (Abcam, ab57602), or 1:250 Rabbit anti-TRAK1 (Atlas Antibodies, HPA005853), or 1:250 Rabbit anti-TRAK2 (Atlas Antibodies, HPA015827), or a combination of Mitofusin 1 with TRAK1 or Mitofusin 1 with TRAK2 antibodies). The remainder of the staining protocol was performed according to the manufacturer's instructions (Duolink® PLA). In brief, coverslips were incubated in a humidity chamber with anti-mouse MINUS and anti-rabbit PLUS probes (Sigma Aldrich) for 1h at 37° C, hybridized for 30 minutes at 37° C, and amplified for 100 mins

at 37° C using the red fluorophore. Coverslips were then allowed to dry at room temperature for 20 mins in the dark, and mounted using Duolink® In Situ mounting medium containing DAPI.

Coimmunoprecipitation

COS7 cells expressing Miro1myc or Miro2myc with either GFP or GFP-Myo19 were homogenized in lysis buffer (50 mm HEPES; 0.5% Triton X-100; 150 mM NaCl; 1 mM EDTA; 1 mM PMSF; and 1 µg/ml antipain, pepstatin, and leupeptin; pH7.5). Cell debris was cleared by centrifugation at 12,000g for 10 minutes. Lysates were incubated for 2 hours with GFP-trap beads (Chromotek). At the end of the incubation the agarose beads were subjected to 4 washes with lysis buffer and resuspended in Laemmli buffer prior to loading in acrylamide gels.

Image acquisition and analysis (extended procedures)

Confocal imaging. Confocal images (1024 x 1024 pixels) were acquired on a Zeiss LSM700 upright confocal microscope (Carl Zeiss, Welwyn Garden City, UK) using a 63X oil immersion objective (NA: 1.4). Images were processed with ImageJ software (http://imagej.nih.gov/ij/). Mitochondrial morphology was scored from MitoTracker stained cells by a blinded researcher. Data collected from three independent experiments (number of cells scored: WT 633; Miro1^{KO} 517; Miro2^{KO} 542 and Miro^{DKO} 455). Two different cells lines per genotype were used in each experiment.

PLA image analysis. Confocal images were taken from fields of view that were selected based on DAPI staining, ensuring each image contains a similar amount of cells to avoid bias. Images were captured using ZEN software with excitation 405 nm for DAPI, and 555 nm for the PLA red fluorophore (equivalent to Texas Red). The pinhole was set to 1.5 Airy units creating an optical slice of 1.2 um. Image stacks of 4 slices were acquired with voxel dimensions of 0.199 x 0.199 x 0.578 μm^3. Analysis was performed with ImageJ. A threshold was selected for DAPI and PLA signal and kept constant for

all data sets. PLA dots and nuclei were then counted using an automated ImageJ script. Staining was performed 3 times. In each experiment, 7 images per condition were acquired, and the results averaged. The averages from conditions with single antibodies were summed to calculate a theoretical background for the combined antibodies in the absence of interaction.

Myo19 and GFPMyo19 signal quantification. MEF cells from the different genotypes were processed at the same time and imaged using the same acquisition parameters. In all images background was subtracted (ImageJ) using the same settings. Mitochondrial signal (MitoTracker) was thresholded to produce a region of interest (ROI) from the mitochondria. Then this area was subtracted from the total cell area to give a cytoplasmic ROI. The ratio between mitochondrial mean intensity divided by cytoplasmic mean intensity generated the mitochondrial enrichment value used throughout the paper.

Mitochondrial transport: MEFs cells expressing MtdsRed2 or LysoGFP were imaged maintaining a constant flow of 36.5°C imaging buffer (10mM HEPES, 125mM NaCl, 10mM D-Glucose, 5mM KCl, 2mM $CaCl_2$ and 1mM $MgCl_2$ at pH7.4). Movies were generated taking one frame every 5 seconds for 5 minutes (61 frames). Generated movies were then processed with ImageJ. Alignment (stackreg) and background subtraction were applied when required. Mitochondrial (and lysosomal) displacement was quantified as previously described (Saotome et al., 2008) with some modifications: mitochondria were thresholded in all frames and the area subtracted with the mitochondrial area of a previous time-point (-10 seconds). The resultant area is the fraction of mitochondrial mass that changed position during the time between frames. This process was repeated with all the pairs of frames (from frame 3 to frame 61) and a single displacement value per cell was averaged. Data was obtained from the indicated number of cells from 6 (mitochondria) or 3 (lysosomes) independent experiments. Two different cell lines per genotype were used per genotype. In the same movies, to quantify microtubule dependent mitochondrial runs we defined this

events as directional displacements of mitochondria that covered at least 5 μm in distance and moved faster than 0.15 μm/sec.

Mitochondrial distribution analysis: MitoTracker was used to stain mitochondria from cells growing on "Y" shaped micropatterned substrates. The acquisition settings to image mitochondria were kept constant across all the images of the experiment and all images were equally processed. The mitochondrial area given by MitoTracker signal was initially defined by a constant threshold value applied equally to all images from the same experiment. Sholl analysis of mitochondrial distribution was performed using a custom made ImageJ plugin (Lopez-Domenech et al., 2016). Mitochondrial signal was quantified within shells radiating out from the soma at 1 μm intervals. The cumulative distribution of mitochondrial signal as a function of the distance from the center of the cell to the periphery was normalized per each cell. An average value was calculated for each distance interval for all the cells in the experiment and a final profile or Mitochondrial Probability Map (MPM) was plotted per genotype. In all plots a theoretical profile (grey dotted line) of a homogeneously distributed signal was generated from a GFP overexpressing cell. The distance at which 50, 90 and 95% of the total mitochondrial mass is found ($Mito^{50}$, $Mito^{90}$ and $Mito^{95}$ values respectively) was calculated per each cell by interpolation. One average $Mito^{95}$ value was calculated per genotype per experiment and used to quantify a final $Mito^{95}$ value (n= number of experiments) in figures. In case of overexpression experiments total number of cells was used as the n (n= number of cells).

Mitochondrial segregation during mitosis. MEFs cells from the indicated genotypes were transfected with either a mitochondrial targeted GFP construct (Su9GFP) or MtDsRed and GFPMyo19 and seeded onto 96-well plates (Ibidi) at a density of 12,000 cells / cm^2. 24 hours later cells were placed on an ImageXpress Micro XLS imaging system (MolecularDevices) with environmental control. Time-lapse movies were taken from 4 different fields per well at a rate of 1 frame every 3 minutes for a total of 10

hours (200 frames). 8 bit images were saved and movies generated with imageJ. After a mitotic event took place a frame where both cells were allowed to completely reattach to the substrate was taken and mitochondria signal was thresholded. From the mitochondrial area the integrated density of the signal was calculated for both cells and the ratio (highest mitochondrial signal / lowest mitochondrial signal) was used as a readout of mitochondrial segregation after mitosis. Data come from at least 3 independent experiments. Two different cell lines per genotype were used.

Appendix Supplementary References

Birsa, N., Norkett, R., Wauer, T., Mevissen, T.E., Wu, H.C., Foltynie, T., Bhatia, K., Hirst, W.D., Komander, D., Plun-Favreau, H., *et al.* (2014). Lysine 27 ubiquitination of the mitochondrial transport protein miro is dependent on serine 65 of the parkin ubiquitin ligase. The Journal of biological chemistry *289*, 14569-14582.

Kilkenny, C., Browne, W.J., Cuthill, I.C., Emerson, M., and Altman, D.G. (2010). Improving bioscience research reporting: the ARRIVE guidelines for reporting animal research. PLoS biology *8*, e1000412.

Lopez-Domenech, G., Higgs, N.F., Vaccaro, V., Ros, H., Arancibia-Carcamo, I.L., MacAskill, A.F., and Kittler, J.T. (2016). Loss of Dendritic Complexity Precedes Neurodegeneration in a Mouse Model with Disrupted Mitochondrial Distribution in Mature Dendrites. Cell reports *17*, 317-327.

Norkett, R., Modi, S., Birsa, N., Atkin, T.A., Ivankovic, D., Pathania, M., Trossbach, S.V., Korth, C., Hirst, W.D., and Kittler, J.T. (2016). DISC1-dependent Regulation of Mitochondrial Dynamics Controls the Morphogenesis of Complex Neuronal Dendrites. The Journal of biological chemistry *291*, 613-629.

Saotome, M., Safiulina, D., Szabadkai, G., Das, S., Fransson, A., Aspenstrom, P., Rizzuto, R., and Hajnoczky, G. (2008). Bidirectional Ca2+-dependent control of mitochondrial dynamics by the Miro GTPase. Proceedings of the National Academy of Sciences of the United States of America *105*, 20728-20733.

Skarnes, W.C., Rosen, B., West, A.P., Koutsourakis, M., Bushell, W., Iyer, V., Mujica, A.O., Thomas, M., Harrow, J., Cox, T., *et al.* (2011). A conditional knockout resource for the genome-wide study of mouse gene function. Nature *474*, 337-342.

Twelvetrees, A.E., Yuen, E.Y., Arancibia-Carcamo, I.L., MacAskill, A.F., Rostaing, P., Lumb, M.J., Humbert, S., Triller, A., Saudou, F., Yan, Z., *et al.* (2010). Delivery of GABAARs to synapses is mediated by HAP1-KIF5 and disrupted by mutant huntingtin. Neuron *65*, 53-65.
