# Supplementary material for: Miro proteins coordinate microtubule‐ and actin‐dependent mitochondrial transport and distribution
Source: EMBO J. 2018 Jan 8;37(3):321–36. doi: 10.15252/embj.201696380 (PMC5793800; doi:10.15252/embj.201696380)
Supplement: Supplementary file 2 — Expanded View Figures PDF [file EMBJ-37-321-s002.pdf]

Expanded View Figures

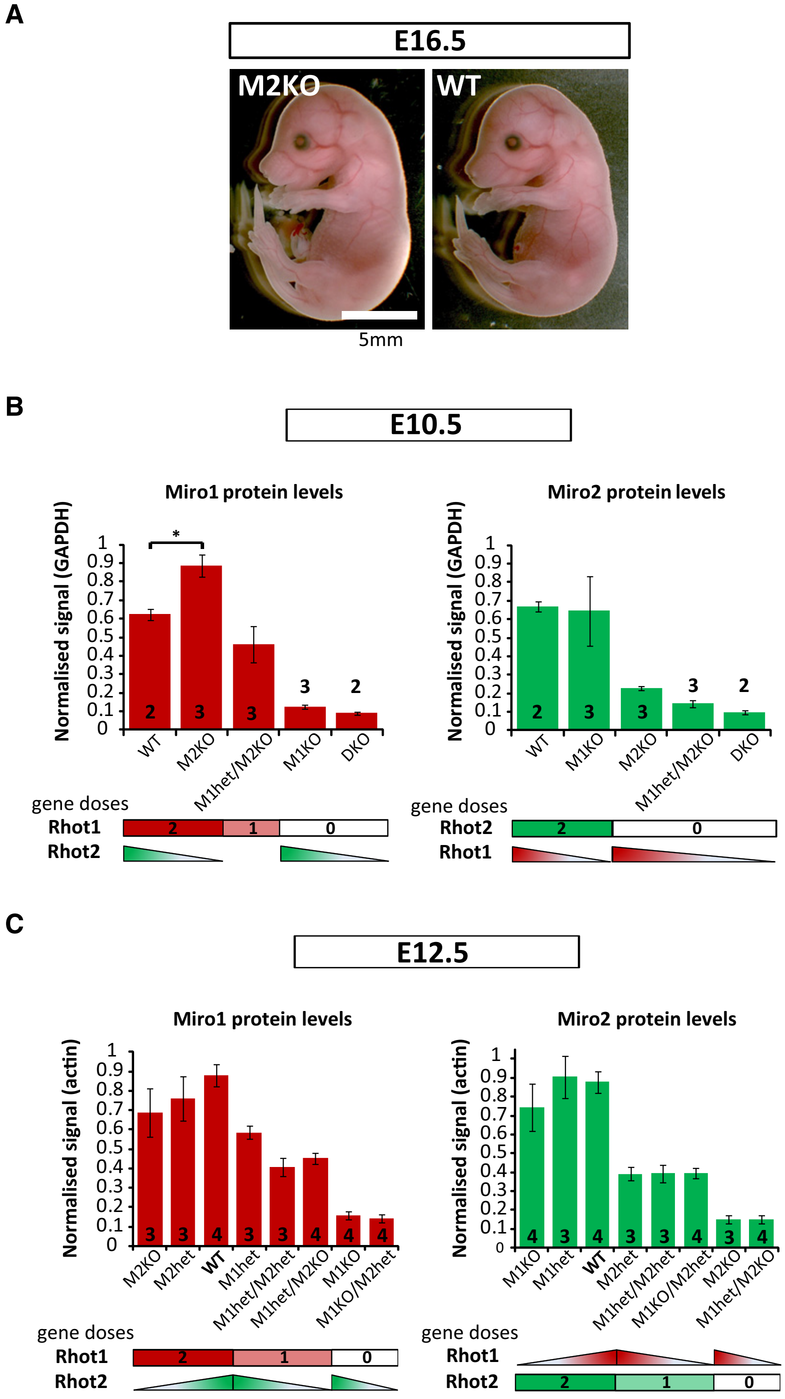

Figure EV1. Role of Miro1 and Miro2 proteins during mouse embryonic development.

A Miro2^{KO} embryos develop normally and are undistinguishable from WT embryos. Image of Miro2^{KO} embryo is taken from Fig 1A.

B, C Quantification of Miro1 and Miro2 protein levels in E12.5 and E10.5 lysates. Miro1 and Miro2 gene doses are stated in the *x*-axis. (B) At E10.5, Miro1 protein shows a ~30% increase in Miro2^{KO} embryos. (C) Miro proteins do not show compensatory mechanisms in E12.5 embryos. All samples were obtained from six pregnant females (at E12.5) and from five pregnant females (at E10.5) and loaded together to allow comparisons (shown in Fig 1F and G). Error bars represent s.e.m. Statistical significance: $*P < 0.05$.

© 2018 The Authors

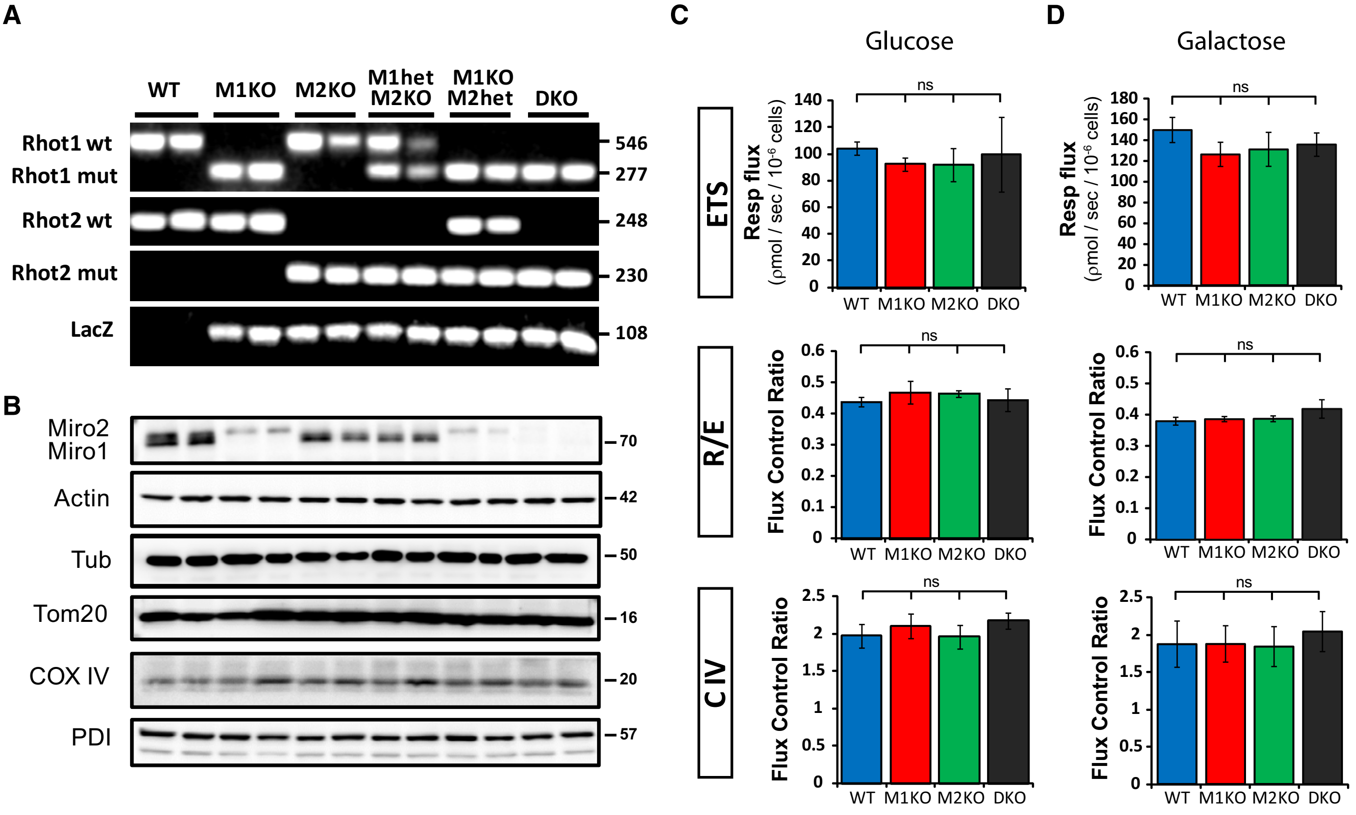

Figure EV2. Genetic and metabolic characterization of Miro1 and Miro2 single and double knockout MEF cell lines.

A, B Genetic (A) and protein (B) characterisation of all MEF cell lines obtained from E8.5 embryos from matings between double heterozygote ($Miro1^{het}$/$Miro2^{het}$) animals.

C Respiration flux of the different MEF cell lines from the main genotypes in standard growing conditions (1,000 mg/l glucose). Units are picomoles of O_2 per second per million cells. Values of electron transfer system (ETS) capacity, routine control ratio (R/ETS) and normalised complex IV activity ratio (C-IV/ETS) are given. Data obtained from three independent experiments (n = 3; ANOVA-NK). In each experiment, respiratory activity was analysed from two different cell lines per genotype. Error bars represent s.e.m.

D Respiratory flux of the different MEF cell lines growing in galactose (15 mM)-supplemented glucose-free medium. Units are picomoles of O_2 per second per million cells. Values of electron transfer system (ETS) capacity, routine control ratio (R/ETS) and normalised complex IV activity ratio (C-IV/ETS) are given. Data obtained from four independent experiments (n = 4; ANOVA-NK). Error bars represent s.e.m.

Source data are available online for this figure.

© 2018 The Authors

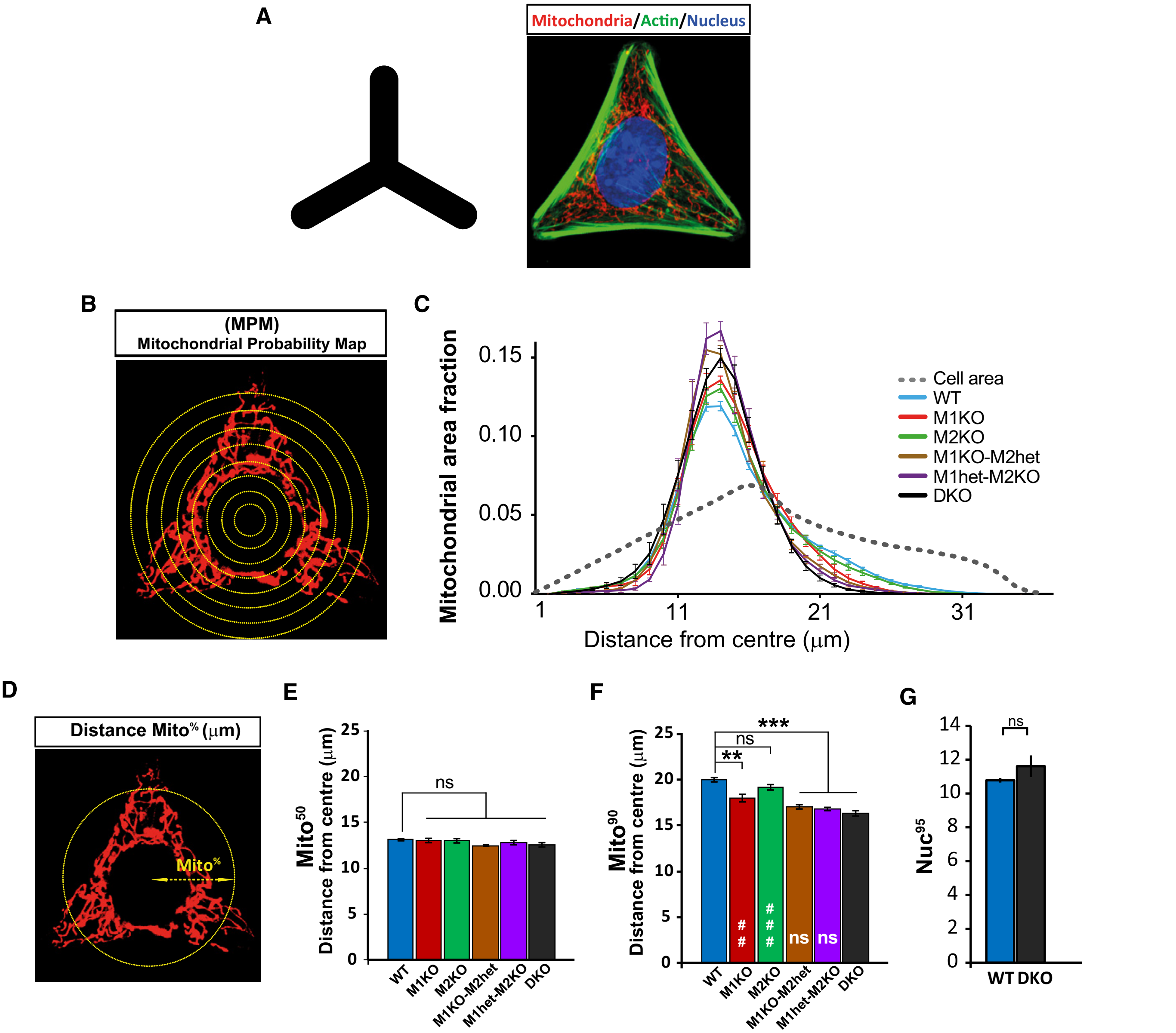

Figure EV3. Representation and quantification of mitochondrial distribution in the different MEF cell lines grown on micropatterned substrates.

A MEF cells were seeded onto "Y"-shaped adhesive micropatterns restricting their growth to an obligate size and shape (triangular) to keep it constant over many cells.

B, C Schematic representation of Sholl analysis of mitochondrial signal (B). Concentric circles growing in diameter from the centre of the cell were used to obtain normalised profiles of mitochondrial distribution (C) in the proximo-distal axis of cells (centre to periphery). Grey dotted line represents the theoretical distribution of a homogeneously distributed signal. The cumulative distribution of these profiles or Mitochondrial Probability Map (MPM) was used to represent the distribution of mitochondria throughout the paper.

D Mito$^{\%}$ values represent the distance from the centre of the cell at which a given fraction of mitochondria is found. All Mito$^{\%}$ values were calculated by interpolation of the mitochondrial signal for each individual cell.

E, F Plotted Mito^{50} (median or 50th percentile) (E) and Mito^{90} (90th percentile) (F) values of the distribution of mitochondrial signal from the different genotypes. Data were obtained from at least three independent experiments (number of experiments: WT 9; Miro1^{KO} 6; Miro2^{KO} 6; Miro1^{KO}/Miro2^{het} 3; Miro1^{het}/Miro2^{KO} 4; Miro^{DKO} 9; ANOVA-NK) where at least 20 cells were analysed per genotype and experiment.

G Nuc^{95} value or distance from the centre of the cell where 95% of nuclear signal is found was calculated and plotted from WT and Miro^{DKO} cells. Data were obtained from three independent experiments. Two different cell lines were used per each genotype.

Data information: Error bars represent s.e.m. Significance: $^{**}P < 0.01$ and $^{***}P < 0.001$.

© 2018 The Authors

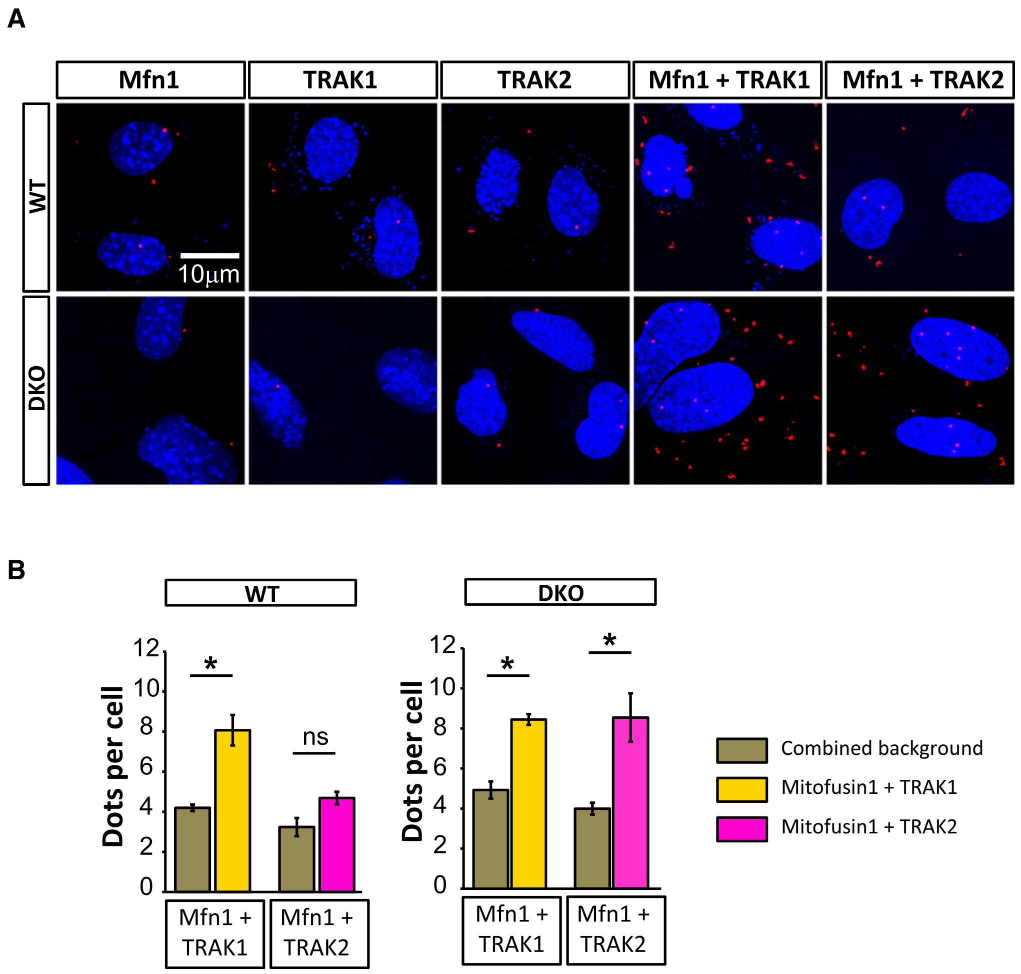

Figure EV4. Mitofusin1 can bind TRAK proteins in the absence of Miro.

A Proximity ligation assay (PLA) showing interaction between endogenous Mfn1 and TRAK proteins as seen by the increased number of fluorescent puncta from the PLA probes (red). DAPI (blue) was used to reveal the nucleus.

B Quantification of the interactions between Mfn1 and TRAK1 and Mfn1 and TRAK2 in both WT and Miro^{DKO} cell lines. Data were obtained from three independent experiments (n = 3; t-test). Error bars represent s.e.m. Statistical significance: *$P < 0.05$.

© 2018 The Authors

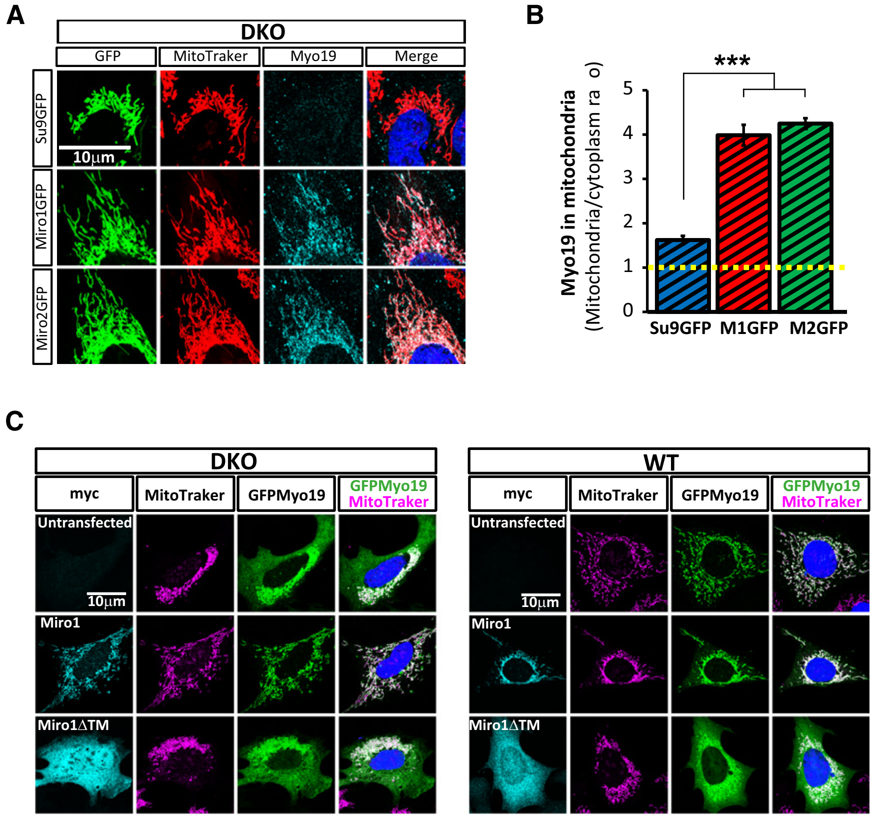

Figure EV5. Miro proteins are able to recruit and stabilize Myo19 to the mitochondria.

A, B Representative images (A) and quantification (B) of endogenous Myo19 signal showing that overexpression of either Miro1GFP or Miro2GFP rescues the levels of Myo19 in mitochondria in Miro^{DKO} cells. Data obtained from 30 cells for each condition from three independent experiments (n = number of cells; ANOVA-NK). Error bars represent s.e.m. Statistical significance: ***$P < 0.001$.

C Miro proteins recruit Myo19 to the mitochondria. Miro^{DKO} and WT cells expressing GFPMyo19 alone or together with the mitochondrial Miro1myc or the cytosolic Miro1ΔTMmyc (see Fig 6I–L for the equivalent experiment with Miro2myc and Miro2ΔTMmyc and quantification).

© 2018 The Authors
