# Supplementary material for: Miro proteins coordinate microtubule‐ and actin‐dependent mitochondrial transport and distribution
Source: EMBO J. 2018 Jan 8;37(3):321–36. doi: 10.15252/embj.201696380 (PMC5793800; doi:10.15252/embj.201696380)
Supplement: Supplementary file 3 — Table EV1 [file EMBJ-37-321-s003.pdf]

Table EV1: Analysis of prenatal viability of embryos from double heterozygotes X double heterozygotes matings. Visual examination and posterior genotyping at the indicated developmental stages.

Stage of development	Number of pups/embryos (number of litters)	Genotype: WT	M1het	M2het	M1het M2het	M1KO	M2KO	M1KO M1het	M1het M2KO	M1KO M2KO	Unknown[a]
**P0**	**27** (5)	4	3	2	13	**1** (0/1)	n.f.	**4** (0/4)	n.f.	n.f.	0
**E16**	**55** (9)	3	3	11	18	3	6	2	**7** (0/7)	n.f.	2
**E14**	**27** (4)	1	1	10	7	n.f.	5	n.f.	**3** (0/3)	n.f.	0
**E12**	**79** (11)	4	13	15	22	5	5	6	**6** (3/3)	n.f.	3
**E10**	**77** (10)	2	9	9	22	6	6	6	**6** (6/0)	**5** (0/5)	6
**E8**	**85** (11)	8	12	9	17	6	5	10	**10** (10/0)	**5** (5/0)	3

Colour coded numbers are (# of embryos viable / # of embryos not viable).

n.f. (not found): Genotype not found at the developmental stage studied.

[a] Unknown genotype due to advanced state of reabsorption.
