# Supplementary material for: Miro proteins coordinate microtubule‐ and actin‐dependent mitochondrial transport and distribution
Source: EMBO J. 2018 Jan 8;37(3):321–36. doi: 10.15252/embj.201696380 (PMC5793800; doi:10.15252/embj.201696380)
Supplement: Supplementary file 5 — Movie EV2 [file EMBJ-37-321-s005.zip › Movie_EV2.rtf]

Movie EV2: Lysosomal displacementLysosomal displacement videos of WT (left) and MiroDKO cells (right). Red labels lysosomal area at any given time. Green represents the new area occupied by lysosomes 10 seconds later. 
