# Supplementary material for: Miro proteins coordinate microtubule‐ and actin‐dependent mitochondrial transport and distribution
Source: EMBO J. 2018 Jan 8;37(3):321–36. doi: 10.15252/embj.201696380 (PMC5793800; doi:10.15252/embj.201696380)
Supplement: Supplementary file 6 — Movie EV3 [file EMBJ-37-321-s006.zip › Movie_EV3.rtf]

Movie EV3: Mitochondrial runsTime lapse video of WT (left) and MiroDKO cells (centre and right) expressing the mitochondrial marker MtDsRed where directional mitochondrial runs can be identified. 
