# Supplementary material for: Miro proteins coordinate microtubule‐ and actin‐dependent mitochondrial transport and distribution
Source: EMBO J. 2018 Jan 8;37(3):321–36. doi: 10.15252/embj.201696380 (PMC5793800; doi:10.15252/embj.201696380)
Supplement: Supplementary file 7 — Movie EV4 [file EMBJ-37-321-s007.zip › Movie_EV4.rtf]

Movie EV4: Mitochondrial segregation during cell divisionLong term imaging example videos of one WT cell (left) and two MiroDKO cells (centre and right) undergoing mitosis. Mitochondria (red) is segregated to daughter cells after mitosis.  
