# Supplementary material for: Miro proteins coordinate microtubule‐ and actin‐dependent mitochondrial transport and distribution
Source: EMBO J. 2018 Jan 8;37(3):321–36. doi: 10.15252/embj.201696380 (PMC5793800; doi:10.15252/embj.201696380)

Figure EV2; Panel B (anti-Miro)

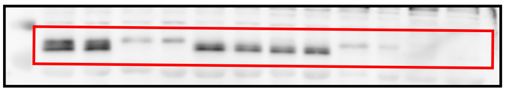

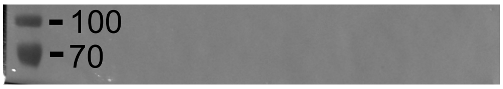

Figure EV2; Panel B (anti-Actin)

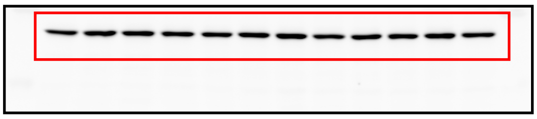

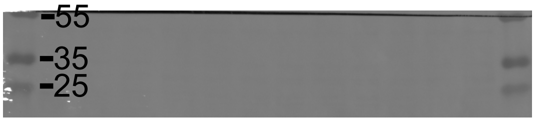

Figure EV2; Panel B (anti-Tubulin)

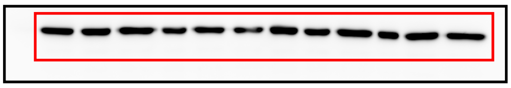

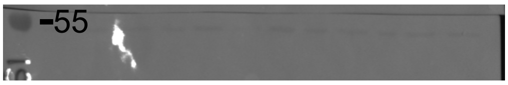

Figure EV2; Panel B (anti-Tom20)

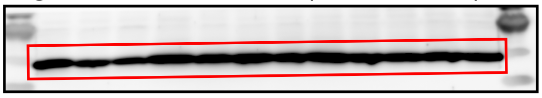

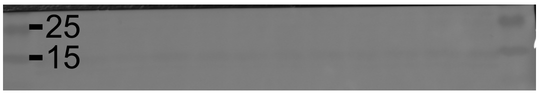

Figure EV2; Panel B (anti-PDI)

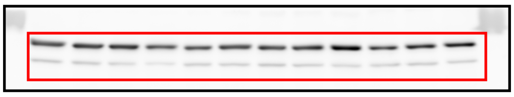

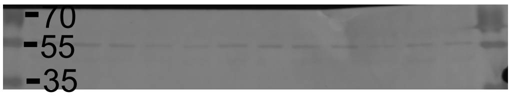

Figure EV2; Panel B (anti-CoxIV)

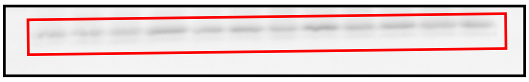

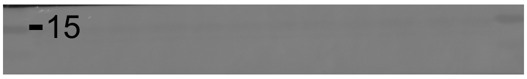

Supplement: Supplementary file 8 — Source Data for Expanded View [file EMBJ-37-321-s012.zip › EMBOJ201696380_Source_Data_FigEV2.pdf]
