# Supplementary material for: Miro proteins coordinate microtubule‐ and actin‐dependent mitochondrial transport and distribution
Source: EMBO J. 2018 Jan 8;37(3):321–36. doi: 10.15252/embj.201696380 (PMC5793800; doi:10.15252/embj.201696380)

Figure 1; Panel F (anti-Miro)

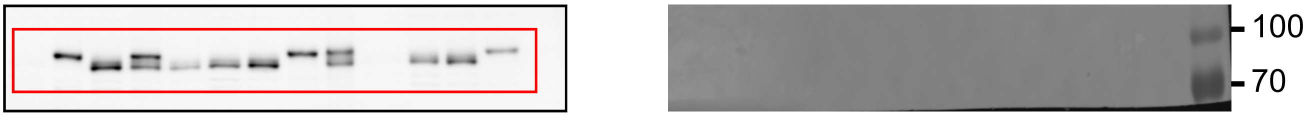

Figure 1; Panel F (anti-PDHE1a / GAPDH / CytC)

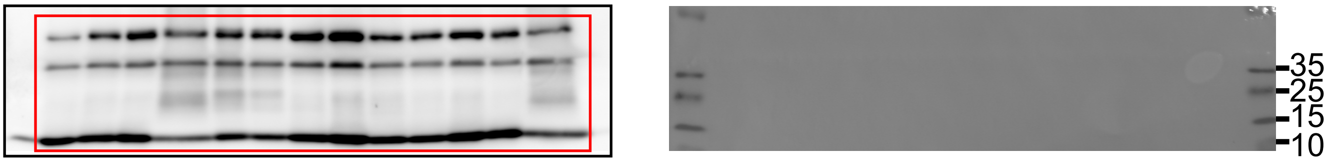

Figure 1; Panel G (anti-Miro)

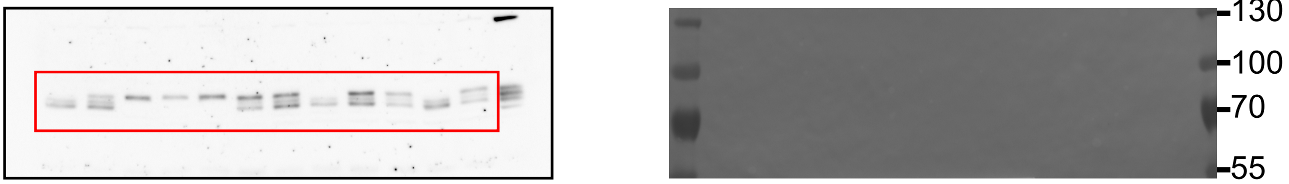

Figure 1; Panel G (anti-Actin)

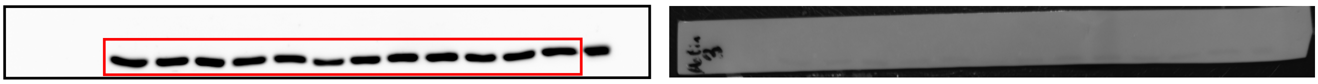

Supplement: Supplementary file 10 — Source Data for Figure 1 [file EMBJ-37-321-s008.pdf]
