# Supplementary material for: Miro proteins coordinate microtubule‐ and actin‐dependent mitochondrial transport and distribution
Source: EMBO J. 2018 Jan 8;37(3):321–36. doi: 10.15252/embj.201696380 (PMC5793800; doi:10.15252/embj.201696380)

Figure 3; Panel E (anti-TRAK1)

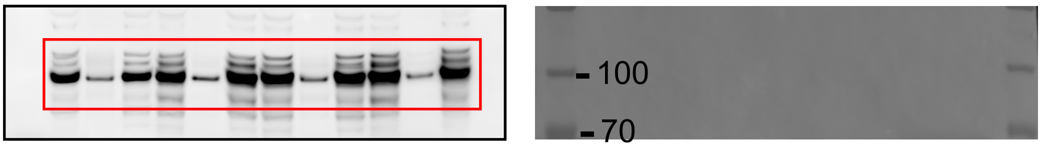

Figure 3; Panel E (anti-TRAK2)

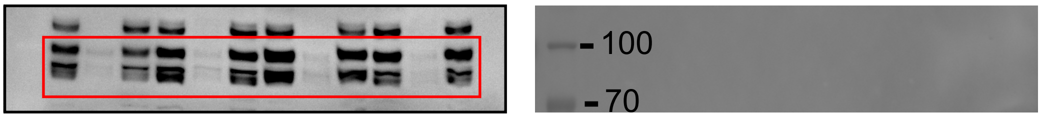

Figure 3; Panel E (anti-KHC)

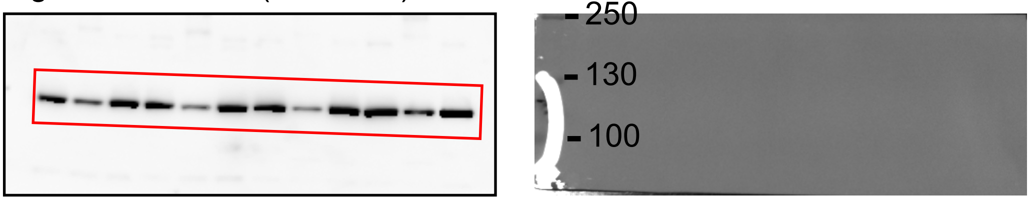

Figure 3; Panel E (anti-p150)

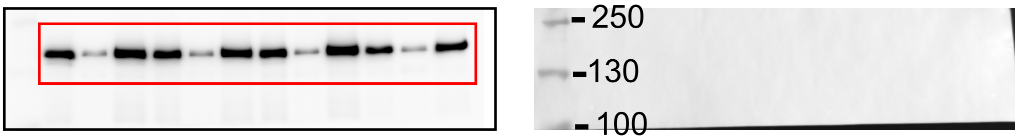

Figure 3; Panel E (anti-DIC)

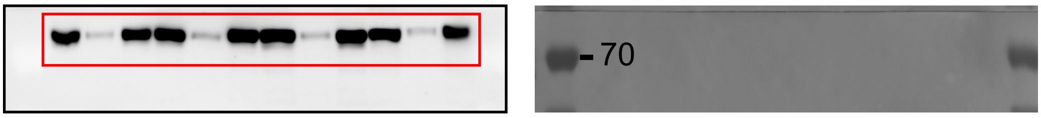

Figure 3; Panel E (anti-Tom20)

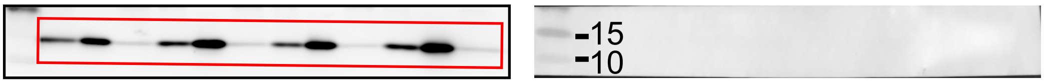

Figure 3; Panel E (anti-GAPDH)

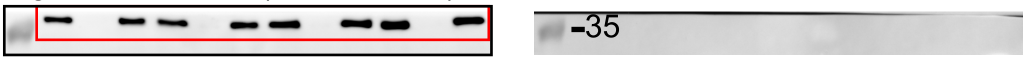

Figure 3; Panel E (anti-CVa/PDHE1a)

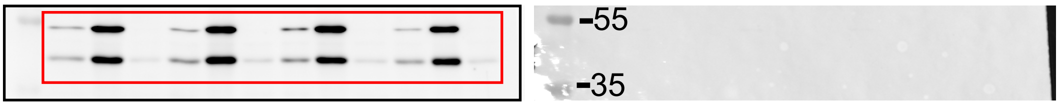

Supplement: Supplementary file 11 — Source Data for Figure 3 [file EMBJ-37-321-s009.pdf]
