# Supplementary material for: Miro proteins coordinate microtubule‐ and actin‐dependent mitochondrial transport and distribution
Source: EMBO J. 2018 Jan 8;37(3):321–36. doi: 10.15252/embj.201696380 (PMC5793800; doi:10.15252/embj.201696380)
Supplement: Supplementary file 12 — Source Data for Figure 6 [file EMBJ-37-321-s010.pdf]

Figure 6; Panel A (anti-Myo19)

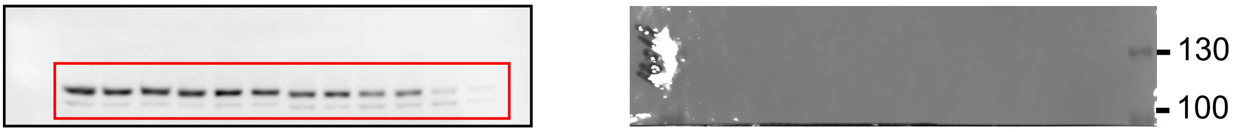

Figure 6; Panel A (anti-Actin)

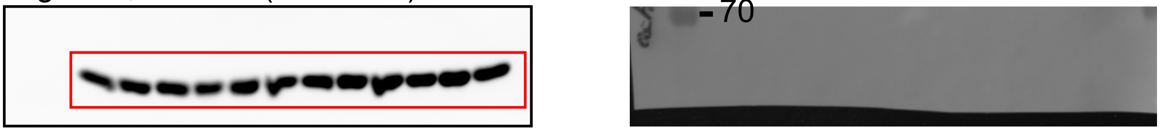

Figure 6; Panel C (anti-Myo19)

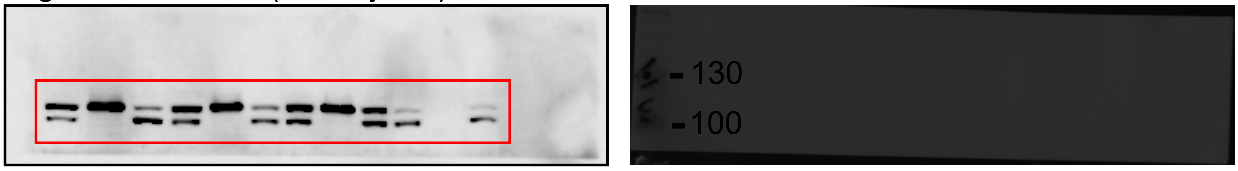

Figure 6; Panel C (anti-CVa/PDHE1a)

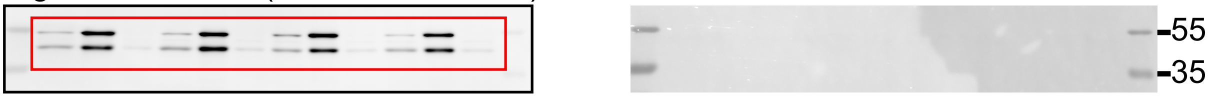

Figure 6; Panel C (anti-GAPDH)

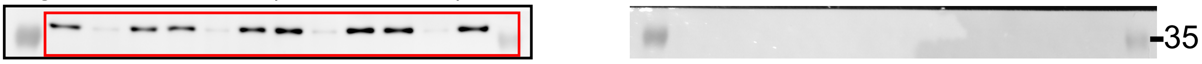

-35

-55

-35
