# Supplementary material for: Miro proteins coordinate microtubule‐ and actin‐dependent mitochondrial transport and distribution
Source: EMBO J. 2018 Jan 8;37(3):321–36. doi: 10.15252/embj.201696380 (PMC5793800; doi:10.15252/embj.201696380)

Figure 7; Panel A

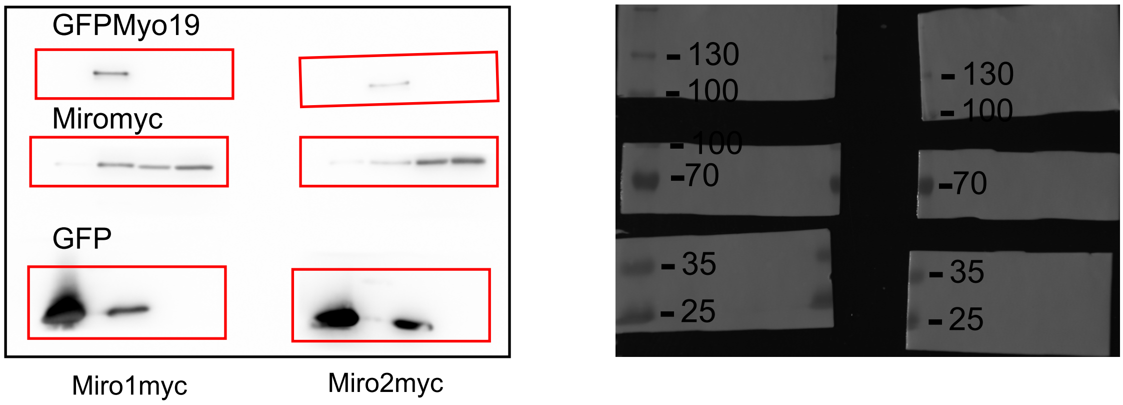

Figure 7; Panel B (anti-Myo19) - WT

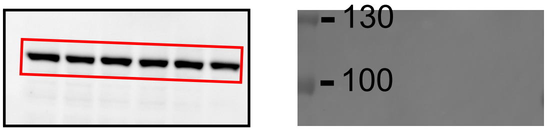

Figure 7; Panel B (anti-actin) - WT

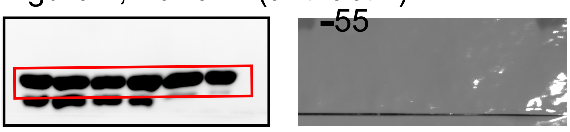

Figure 7; Panel B (anti-Myo19) - M1KO

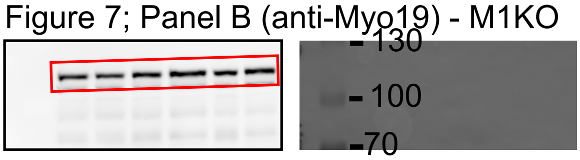

Figure 7; Panel B (anti-actin) - M1KO

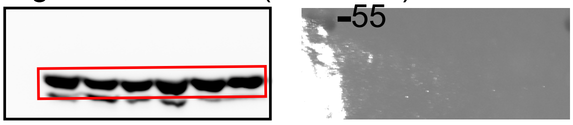

Figure 7; Panel B (anti-Myo19) - M2KO

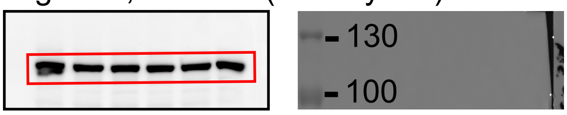

Figure 7; Panel B (anti-actin) - M2KO

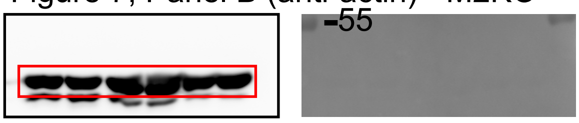

Figure 7; Panel B (anti-Myo19) - DKO

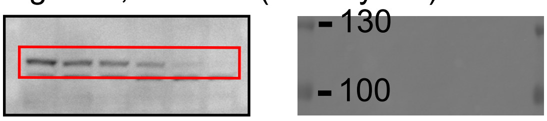

Figure 7; Panel B (anti-actin) - DKO

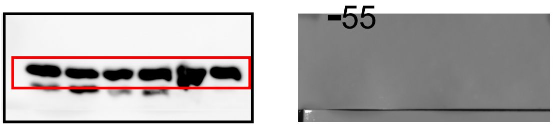

Figure 7; Panel D (anti-Myo19)

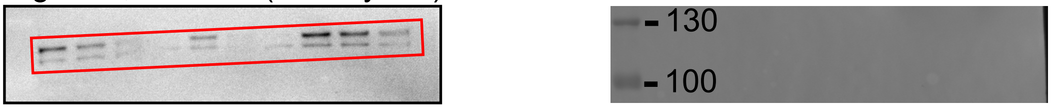

Figure 7; Panel D (anti-actin)

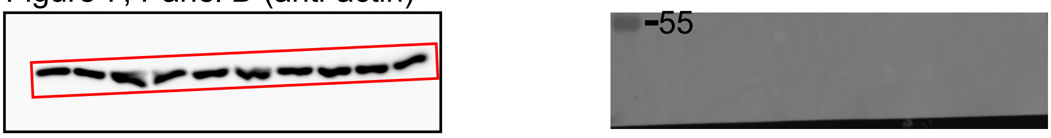

Figure 7; Panel F (anti-Miro1)

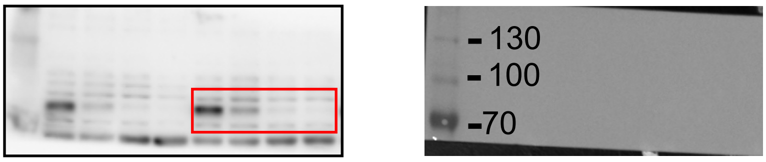

Figure 7; Panel F (anti-Myo19)

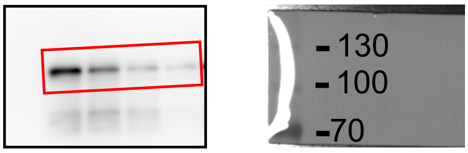

Figure 7; Panel F (anti-PDHE1a)

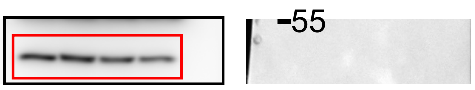

Supplement: Supplementary file 13 — Source Data for Figure 7 [file EMBJ-37-321-s011.pdf]
